# X-ray Diffraction Imaging of Deformations in Thin Films and Nano-Objects

**DOI:** 10.3390/nano12081363

**Published:** 2022-04-15

**Authors:** Olivier Thomas, Stéphane Labat, Thomas Cornelius, Marie-Ingrid Richard

**Affiliations:** 1 Aix Marseille Univ, CNRS, IM2NP UMR 7334, Campus de St-Jérôme, 13397 Marseille, France; stephane.labat@univ-amu.fr (S.L.); thomas.cornelius@im2np.fr (T.C.); mrichard@esrf.fr (M.-I.R.); 2 ID01/ESRF, The European Synchrotron, 71 Rue Des Martyrs, 38043 Grenoble, France

**Keywords:** X-ray diffraction, strain, mapping, nanostructures

## Abstract

The quantification and localization of elastic strains and defects in crystals are necessary to control and predict the functioning of materials. The X-ray imaging of strains has made very impressive progress in recent years. On the one hand, progress in optical elements for focusing X-rays now makes it possible to carry out X-ray diffraction mapping with a resolution in the 50–100 nm range, while lensless imaging techniques reach a typical resolution of 5–10 nm. This continuous evolution is also a consequence of the development of new two-dimensional detectors with hybrid pixels whose dynamics, reading speed and low noise level have revolutionized measurement strategies. In addition, a new accelerator ring concept (HMBA network: hybrid multi-bend achromat lattice) is allowing a very significant increase (a factor of 100) in the brilliance and coherent flux of synchrotron radiation facilities, thanks to the reduction in the horizontal size of the source. This review is intended as a progress report in a rapidly evolving field. The next ten years should allow the emergence of three-dimensional imaging methods of strains that are fast enough to follow, *in situ*, the evolution of a material under stress or during a transition. Handling massive amounts of data will not be the least of the challenges.

## 1. Introduction

The quantification and localization of elastic deformations and defects in crystals are necessary to control and predict the functioning of materials. This is obvious when it comes to structural materials, wherein it is essential to know, for example, the places where the mechanical stresses are concentrated. At small scales, it is now well established that materials can be elastically deformed to much greater extents (1% and beyond) than bulk materials can sustain. This size effect has given new life to the field of stress engineering, which consists of modifying the properties of materials by deforming them. Thus, the mobility of the charge carriers in the transistors of our electronic devices is increased by the controlled and very local application (the size of the transistors today is only 10 nm) of a mechanical stress [1]. Many other physical properties, such as magnetic anisotropy [2], optical properties [3], catalytic activity [4], etc., can be modified by the deformation of the crystal lattice. In all these examples, it is preferable to map the deformations of the crystal lattice with the best possible sensitivity and spatial resolution. From this point of view, X-ray diffraction has undeniable advantages: high sensitivity to lattice deformations, an open environment allowing access to numerous diffracting planes and consequently to the complete strain tensor, low absorption allowing the analysis of buried materials without special preparation, and a non-destructive character.

The diffraction of X-rays by crystals was demonstrated for the first time by M. Laue [5] in 1913, using a polychromatic X-ray beam and photographic film. Shortly after Laue’s discovery, Laue diagrams that were recorded from deformed crystals were observed to show elongated spots called asterism [6,7]. Thus, the sensitivity of X-ray diffraction to crystal imperfections was realized even before the concept of dislocation was introduced independently by Taylor [8], Polanyi [9] and Orowan [10,11,12] at the beginning of the 1930s to account for the plastic deformation of solids. The elastic strains and the associated stresses can be deduced with great precision from the shift of the diffraction peaks [13,14]. This approach remains very effective for measuring elastic strains in various materials [15].

Imaging of deformations with X-rays has made very impressive progress in recent years. On the one hand, advances in X-ray-focusing optical elements now make it possible to perform X-ray diffraction mapping with a resolution in the 50–100 nm range [16,17]. Full-field X-ray microscopy has also improved a great deal, with resolutions of the order of 100 nm [18]. By far the best spatial resolution is obtained with coherent diffraction imaging, which is a lensless imaging technique, with a typical resolution of 5–10 nm [19,20].

This continuous evolution is also a consequence of the development of new two-dimensional hybrid pixel detectors [21,22,23] whose dynamic range, reading speed and low noise level have revolutionized measurement strategies. In addition, the year 2020 has seen the starting up of the new ring of the ESRF synchrotron facility, called EBS (Extremely Brilliant Source). This new concept (HMBA lattice: a hybrid multi-bend achromat lattice), already used in part for the Swedish MAX IV source, will allow a very significant increase (a factor of 100) in brilliance and coherent flux, thanks to the reduction in the horizontal size of the source. Most synchrotrons around the world have upgrade programs intended to take advantage of this new type of source.

Thus, this article is very modestly intended as a stepping-stone in a rapidly evolving field. We have no doubt (we even hope!) that part of this manuscript will quickly become obsolete, but the fundamental approaches will probably remain unchanged. On the other hand, the next ten years should allow the emergence of three-dimensional imaging methods of deformations that are fast enough to follow *in situ* the evolution of a material under stress or during a transition. Processing huge amounts of data will not be the least of the challenges.

After briefly outlining the elements necessary for the understanding of X-ray diffraction, we present the methods available today to map the deformations of the lattice and we present some recent examples of the use of these methods. We limit ourselves here to the study of single-crystal materials, but some of the methods that we describe can be applied to polycrystals where the grain size is large enough.

## 2. X-ray Diffraction by a Distorted Crystal

### 2.1. Kinematic Theory—Reminder

Most X-ray photons are absorbed by condensed matter through the photoelectric effect. Only a tiny proportion is elastically scattered (Thomson scattering). This very weak interaction is basically an advantage because—in the context of Born’s first approximation, which is generally called the kinematic approximation in the field of X-ray diffraction—it makes it possible to write out the scattered amplitude as the Fourier transform of electron density [24,25]:

(1) $$A(\vec{q})=\int \rho (\vec{r})e^{i\vec{q}.\vec{r}}d\vec{r}$$

where $\rho (\vec{r})$ is the electron density and $\vec{q}={\vec{k}}_{diff}-{\vec{k}}_{in}$ is the scattering vector, defined as the difference between the incident wave vector and the scattered wave vector. We note the angle between ${\vec{k}}_{diff}$ and ${\vec{k}}_{in}$ conventionally as 2θ. It follows that:

(2) $$q=4\pi \frac{sin\theta }{\lambda }$$

where *λ* is the wavelength.

Equation (1) remains an approximation and, in the case of perfect crystals, must be justified. In general, crystals where the dimensions are smaller than the extinction length diffract in the kinematic regime [26].

In the case of an undistorted crystal of finite size and whose shape function is $s(\vec{r})$, the scattered amplitude becomes:

(3) $$A(\vec{q})=TF\left\{s(\vec{r})\underset{m}{\sum }\rho_{c}(\vec{r})*\delta (\vec{r}-\vec{R_{m}})\right\}=S(\vec{q})*F(\vec{q})\underset{m}{\sum }e^{i\vec{q}.\vec{R_{m}}}$$

where $\vec{R_{m}}$ is a Bravais lattice vector, $\rho_{c}(\vec{r})$ is the electron density in the unit cell, $F(\vec{q})$ is its Fourier transform, called the structure factor, $S)\vec{q})$ is the Fourier transform of $s(\vec{r})$, δ is the Dirac distribution, and * represents the convolution product. This expression brings up a Dirac comb function:

(4) $$A(\vec{q})=S(\vec{q})*\frac{F(\vec{q})}{V}\underset{m}{\sum }\delta (\vec{q}-\vec{G_{m}})$$

where ${\vec{G}}_{m}$ is a vector of the reciprocal lattice and V is the volume of the unit cell. This well-known expression shows that diffraction by an undistorted crystal gives rise to well-defined diffraction peaks in space at positions $\vec{q}={\vec{G}}_{m}$ (Laue condition). It is easy to show that this is equivalent to Bragg’s law:

(5) $$2d_{hkl}sin\theta =\lambda$$

where *d_hkl_* is the interplanar spacing for (*hkl*) planes.

What happens in the case of a deformed crystal? If the deformation is homogeneous in the volume probed by the X-ray beam (a volume that, under current conditions, can be quite small (see Section 3.1)) then the deformed cell is repeated periodically and the determination of the new positions of the diffraction peaks allows us to determine the unit cell parameters that can be compared with those of the undeformed cell. This is the simplest situation. The ability of X-ray diffraction to determine crystalline parameters with very high precision makes it the tool of choice for the quantitative determination of deformations from the position of diffraction lines. This point is dealt with in Section 2.2.

If the deformation is not homogeneous in the probed volume, then the shape of the diffraction peaks will be modified. The possibility of quantifying the displacement field inside the crystal will depend on several factors, such as the coherence of the beam, the nature of the distribution of displacements, etc. The growing mastery of imaging approaches based on coherent diffraction has enabled major advances in recent years. This point is addressed in Section 2.3.

### 2.2. Analysis of Diffraction Peak Displacements

A spatially homogeneous deformation—at the scale of the beam—of the crystal lattice will give rise to a displacement of the diffraction peaks. This displacement is directly related to the variation of the crystalline parameters via Bragg’s law, which it suffices to differentiate:

(6) $$\Delta 2\theta_{hkl}=2\;tan\theta_{hkl}\;\frac{\Delta d_{hkl}}{d_{hkl}}$$

Thus, a relative inter-reticular distance variation of 0.1% gives rise to a displacement of approximately 0.1° at 2θ = 90° and 0.02° at 2θ = 20°. We recognize here the greater sensitivity to deformations at large diffraction angles that is always preferable for use when the experimental conditions allow it. The measurement of the diffraction angle corresponds to a radial scan in the reciprocal space. Today, with the increasingly systematic use of two-dimensional detectors, it is becoming common (and faster) to make three-dimensional maps of the reciprocal lattice. The location of the center of gravity of the measured node makes it possible to completely determine the scattering vector $\vec{q}$: its modulus is linked to the variation of the diffraction angle 2θ and its orientation in space, is linked to the rotation of the reticular planes in the distorted crystal.

**Figure 1:**
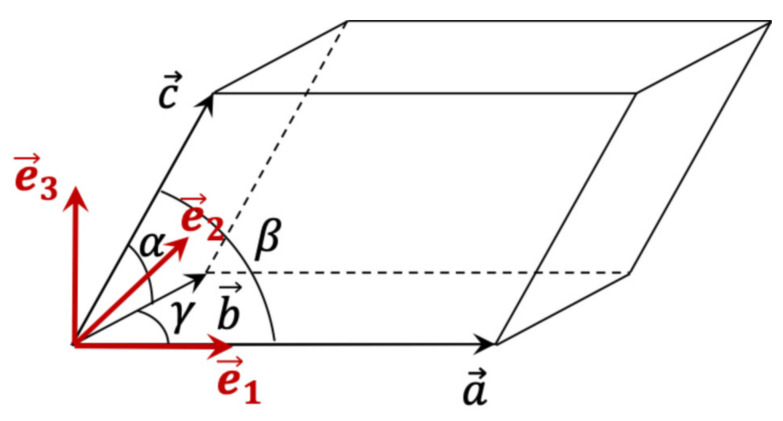
Orthogonal reference basis associated with the unit cell.

Whatever the origin of the (homogeneous) deformation of the lattice, its physical interpretation requires the use of an orthonormal reference basis $\left(\vec{e_{1}},\vec{e_{2}},\vec{e_{3}}\right)$ attached to the unit cell. There are several conventions [27,28,29] to define this reference frame, regarding which the elastic, thermal, piezoelectric strains, etc., are expressed. In the same way, the anisotropic properties of the crystal (tensor of the constants of elasticity, for example) are expressed in this frame of reference. In the convention recommended by [28,29], the orthonormal frame is defined as follows (Figure 1):

(7) $$\vec{e_{1}}=\frac{\vec{a}}{a},\;\;\;\;\vec{e_{2}}\;\mathrm{in}\;\;(\vec{a},\vec{b})\;\mathrm{plane}\;\mathrm{and}\;\vec{e_{3}}=\vec{e_{1}}\wedge \vec{e_{2}}$$

In the case of monoclinic or triclinic lattices, it is necessary to check what convention is being used. For example, Nye [27] recommends $\vec{e_{2}}=\frac{\vec{b}}{b}$ in the monoclinic case. In the Busing convention [28], the transformation matrix from the cell frame to the orthonormal frame is:

(8) $$B=\left(\begin{array}{ccc} a & b\;cos\gamma & c\;cos\beta \\ 0 & b\;sin\gamma & -csin\beta cos\alpha^{*}\\ 0 & 0 & 1/c^{*} \end{array}\right)$$

where the “starred” terms designate the unit cell parameters in the reciprocal lattice.

In continuum physics, the deformation of a solid is based on the displacement field, which describes the displacement of each point of the solid after deformation. In the case of a crystal, the crystalline elasticity describes the deformation of the lattice [30] but not that of the basis. The deformation of the basis under the influence of a mechanical stress is a phenomenon that is less frequently addressed [31,32]. However, it is essential to describe the evolution of electronic properties under stress, for example, in semiconductors. Note in this case that the deformation of the basis is deduced from the intensity of the weak or forbidden lines and not from their position.

Let $\vec{u_{m}}$ be the displacement of the node, m, in the deformed crystal, with respect to the undeformed crystal: $\vec{R_{m}^{\prime }}=\vec{R_{m}}+\vec{u_{m}}$. In the small displacement approximation, the deformation of the crystal is described by the displacement gradient tensor **e**, the 9 components of which are written: $e_{ij}=\frac{\partial u_{i}}{\partial x_{j}}$. Thus: $u_{i}=u_{0i}+e_{ij}x_{j}$, where $\vec{u_{0}}$ is the displacement of the origin.

The displacement gradient tensor **e** can be broken down into a symmetric part **ε**, which represents the deformation, and an antisymmetric part **ω**, which represents the rotation:

**e = ε + ω** (9)

(10) $$\varepsilon_{ij}=\frac{1}{2}\left(\frac{\partial u_{i}}{\partial x_{j}}+\frac{\partial u_{j}}{\partial x_{i}}\right)\;$$

(11) $$\omega_{ij}=\frac{1}{2}\left(\frac{\partial u_{i}}{\partial x_{j}}-\frac{\partial u_{j}}{\partial x_{i}}\right)\;$$

The parameter that can be extracted by diffraction is the displacement field (distances are measured) and a derivation operation is always necessary to deduce the displacement gradient. The measurement of a distance *d_hkl_* can always be reduced to a strain from a reference distance (taken at time t = 0, or at temperature T_0_, or in a zone considered “reference”, even in a substrate of a different nature, etc.). On the other hand, it is often necessary to determine the elastic strains that make it possible to determine the stresses using Hooke’s law:

(12) $$\sigma_{\mathrm{ij}}=C_{\mathrm{ijkl}}\varepsilon_{\mathrm{kl}}$$

It is then essential to know the stress-free unit cell parameters. It is sometimes possible to overcome these data, which are always difficult to obtain with the required precision, by relying on the symmetry of the stress tensor. Thus, in the case of a biaxial stress state (x_1_,x_2_ plane) in a cubic and elastically isotropic crystal, it is easy to show [15] that:

(13) $$\frac{a_{\psi }-a_{0}}{a_{0}}=\frac{1+\nu }{E}\sigma_{\varphi }sin^{2}\psi -\frac{\nu }{E}\left(\sigma_{11}+\sigma_{22}\right)$$

where $a_{\psi }$ and $a_{0}$ are, respectively, the unit cell parameters at inclination ψ and free of stress. E and ν are the Young’s modulus and Poisson’s ratio, respectively. φ is the azimuth in the plane (x_1_, x_2_) and σ_ij_ is the stress. Moreover, the stress in the plane at one azimuth φ is:

(14) $$\sigma_{\varphi }=\sigma_{11}cos^{2}\varphi +\sigma_{12}sin2\varphi +\sigma_{22}sin^{2}\varphi \;$$

**Figure 2:**
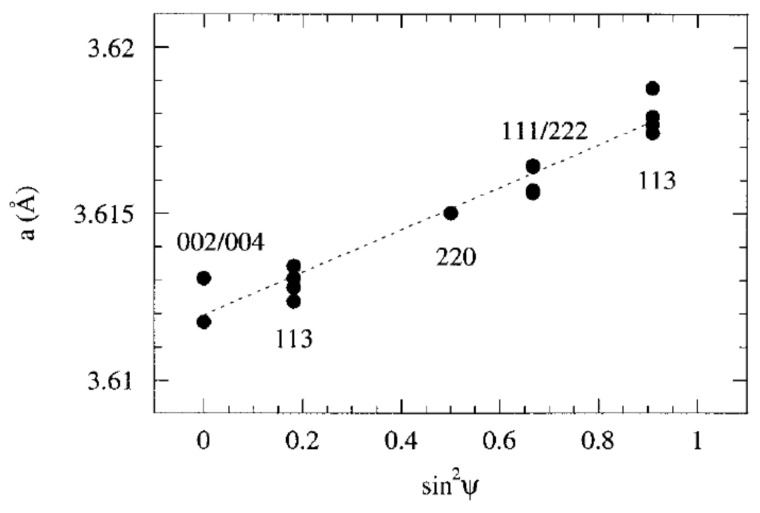
The Sin2ψ law in a monocrystalline Cu film with an orientation of (001) deposited on silicon [33]. The positive slope is proportional to the residual tensile stress present in the film. Reprinted from [33].

Thus, the evolution of *a* as a function of *sin^2^ψ* is linear and the slope gives direct access to the stress. The exact knowledge of *a_0_* is not necessary for the first order and one can choose *a_0_* = *a*(ψ = 0) as an example. Figure 2 shows such a representation *a*(ψ) for a monocrystalline film of copper on silicon [33]. The positive slope highlights a residual tensile stress in the plane of the film.

In the case of thin films or even supported nanostructures, the temperature evolution of the lattice parameter provides information on the possibly thermoelastic nature of the mechanical stress in the film. If we consider a film of elastically isotropic material (Young’s modulus E and Poisson’s ratio ν) and the isotropic expansion coefficient (α_f_) deposited on a substrate with an expansion coefficient α_s_, the effective expansion coefficient of the film becomes anisotropic and equals:

(15) $$\alpha_{eff}^{⊥}=\frac{1+\nu }{1-\nu }\alpha_{f}-\frac{2\nu }{1-\nu }\alpha_{s}\;$$

(16) $$\alpha_{eff}^{∕∕}=\alpha_{s}\;$$

Thus, the expansion coefficient in the perpendicular direction can be significantly increased by the Poisson effect. In the common case of a Poisson’s ratio, $\nu =\frac{1}{3}$, we obtain:

(17) $$\alpha_{eff}^{⊥}=2\alpha_{f}-\alpha_{s}.$$

For a metallic film on a substrate with a low coefficient of thermal expansion, the coefficient of expansion is almost doubled.

**Figure 3:**
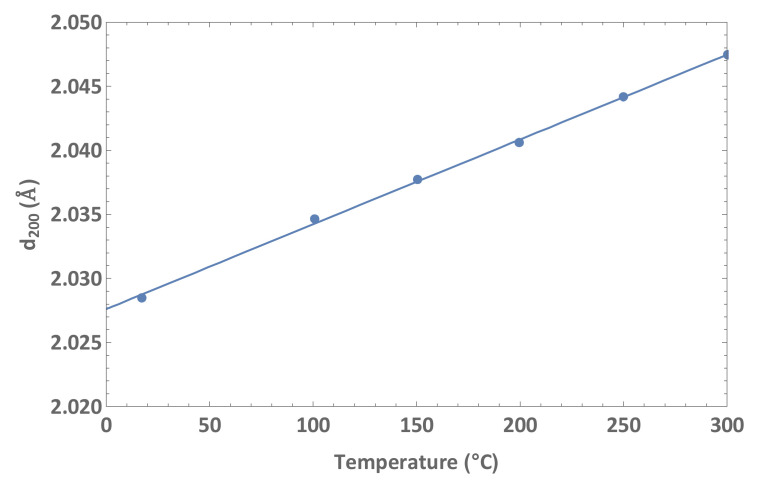
Distance between (200) planes parallel to the surface, as a function of temperature, in a gold film deposited on silicon. The Poisson effect gives rise to an effective coefficient of thermal expansion that is more than twice the coefficient of free expansion.

Figure 3 shows the evolution of the distance between (200) planes in a gold film ($\alpha_{f}=14.2\times 10^{-6}\;K^{-1}$) deposited on silicon [34]. Linear regression gives $\alpha_{eff}^{⊥}=32.5\times 10^{-6}\;K^{-1}$, which is in good agreement with the prediction for a monocrystalline film with an orientation of <100>:

(18) $$\alpha_{eff}^{⊥}=\frac{\left(C_{11}+2C_{12}\right)\alpha_{f}-2C_{12}\alpha_{s}}{C_{11}}≅34\times 10^{-6}\;K^{-1}$$

### 2.3. Diffraction Peak Shape Analysis

Very early on, it was realized that the size of the crystallites contributes to the broadening of the diffraction peaks [35]. The corresponding formalism is quite straightforward when considering kinematic diffraction as a “simple” Fourier transform. The contribution of the displacement field to the broadening of the diffraction lines is, on the other hand, a much more complex problem. The literature devoted to this subject is considerable and there is not enough space here to detail the many approaches to line profile analysis that have emerged since the 1940s, whether Fourier analysis-type approaches [36,37] or those based on the determination of the moments of the profile [38]. A good overview of the different approaches can be found in [39]. In all cases, it is a question of extracting statistical parameters that are representative of the microstructure (microstrain: standard deviation of the strain distribution, the density of dislocations, etc.) from the profile of one or more diffraction lines. Given the complexity of the problem, these methods inevitably rely on strong hypotheses that it is important to be able to test if possible. The use of focused X-ray beams makes it possible to spatially resolve the different contributions to the line profile and sometimes makes it possible to test these macroscopic models. This is how L. Levine and his collaborators at NIST [40] demonstrated the validity of Mughrabi’s hypothesis [41] on the origin of asymmetric line profiles in deformed metals: microbeam measurements have confirmed that dislocation cells are more strongly deformed than neighboring dislocation-poor regions. Without seeking to be exhaustive, we can also cite the fine study by F. Hofmann using Laue micro-diffraction to map the rotational field induced by a single dislocation [42].

The last 20 years have seen a revolution in the information that can be obtained from the “shape” of a diffraction spot. In 1999, John Miao [43] demonstrated the possibility of imaging small islands of gold from the far-field diffraction pattern obtained under coherent illumination. This lensless imaging is based on the algorithmic reconstruction of the phase of the diffracted signal, which is possible if the signal is sufficiently oversampled. While this pioneering experiment was carried out at small angles, in 2001, Ian Robinson demonstrated the possibility of using Bragg diffraction to image small gold crystals [44]. A few years later, the remarkable ability of coherent imaging in Bragg conditions (BCDI: Bragg coherent diffraction imaging) was used to image the displacement field in a crystal [19,20]. This sensitivity to atomic displacements in the crystal is very great and can be understood formally as follows. The amplitude scattered by the crystal in the presence of a displacement field $\vec{u}$ is written, as in Equation (3):

(19) $$A(\vec{q})=S(\vec{q})*F(\vec{q})\underset{m}{\sum }e^{i\vec{q}\cdot \vec{u_{m}}}e^{i\vec{q}\cdot \vec{R_{m}}}S(\vec{q})*F(\vec{q})\underset{m}{\sum }e^{i\vec{G}\cdot \vec{u_{m}}}e^{i\vec{q}\cdot \vec{R_{m}}}$$

where $\vec{G}$ is the Bragg vector of the considered reflection and where we made the approximation [45]: $\vec{q}\cdot \vec{u}≅\vec{G}\cdot \vec{u},$ verified on condition of $\vec{q}$ being close to $\vec{G}$ (i.e., staying in the vicinity of the diffraction peak), and provided that the displacement is not too large. Thus, the amplitude scattered by the crystal appears as the Fourier transform of the complex number $\rho (\vec{r})e^{i\vec{G}\cdot \vec{u}}$. It is the phase of this number that contains all the information on the displacement field, whereas $\rho (\vec{r})$ essentially contains the information on the shape of the crystal in the case of a chemically homogeneous object. This term, $\vec{G}\cdot \vec{u}$, is well known in diffraction theory [25] and implies in particular the classical condition $\vec{G}\cdot \vec{b}\neq 0$ for the visibility of a dislocation. The phase shift of π that a dislocation can introduce leads to a splitting of the diffraction peak into two lobes (of equal amplitude if the defect is at the center of the diffracting volume) as shown experimentally by Jacques et al. [46,47,48] for Frank loops in silicon. Since this pioneering work, many articles have been published, demonstrating the imaging of defects in three dimensions by BCDI [49,50,51,52,53].

## 3. Recent Developments: Toward the 3D Imaging of Deformations

### 3.1. Focusing/Small Beams

The focusing of an X-ray beam depends on the size of the source and the ratio between the optical–focal point distance and the source–optical distance. In a synchrotron installation, the size of the source is typically of the order of a few tens of microns. A magnification ratio of the order of 10^−3^ is, therefore, needed to reduce the size of the beam to a few tens of nanometers. Furthermore, it is preferable not to reduce the optical-sample distance too much, to leave some room for a specific environment and/or the possibility of moving the sample in space. A good compromise is a distance of the order of 10 cm, which then gives a source–optical distance of 100 m. One can clearly see the advantage of building long beamlines, as has been conducted over the past twenty years on many synchrotrons.

The refractive index of condensed matter in the X-ray energy domain is very close and is less than 1 (typically, 1–10^−5^). This makes it difficult to produce refractive lenses but opens up the possibility of focusing optics in reflection, thanks to the phenomenon of total reflection. The three main types of optics developed in recent years are: (1) refractive optics; (2) diffractive optics; (3) reflective optics.

#### 3.1.1. Refractive Optics

Because of the very low refractive index, it is necessary to stack many lenses to obtain a sufficiently small focal length (of the order of 1 m). It is, therefore, necessary to use a weakly absorbing material (Be, B, C, Al, Si). These CRLs (compound refractive lenses) [54,55] are very versatile and are well suited to assemblies requiring a long focal length.

#### 3.1.2. Diffractive Optics

These are mainly systems based on Fresnel zone plates [56]. They consist of a succession of concentric rings that are absorbent (from heavy metals such as Au or W) and transparent (most often, Si) where the radius varies, such as the root of the ring number. According to the theory developed by Augustin Fresnel, the focal length is directly proportional to the width of the outer Fresnel zone. Advances in FZPs (Fresnel zone plates) in recent years have been directly linked to advances in lithography [57]. We are now reaching the limits of the capabilities of lithography, and a new type of lens, based on the same physical principle, has appeared: MLLs (multilayer Laue lenses) or Laue lenses [58]. Fresnel zones are produced by the successive (and not periodic) deposition of thin layers by condensation. Once the deposit has been made (it typically takes a week, which poses formidable stability problems), the lens is cut out and mounted on the edge. This type of lens is the most promising for reaching beam sizes of the order of or less than 10 nm [59].

#### 3.1.3. Focusing Mirrors

These are based on the total reflection of X-rays below the critical angle (of the order of mrad). The configuration that is most used is that imagined by Kirkpatrick and Baez [60,61], which reduces astigmatism. These are two elliptical mirrors mounted perpendicularly. The quality of the focusing of these “KB” mirrors is linked to the mastery of surface polishing (roughness and slope errors). This technology has made steady progress in recent years. The enormous advantage of reflective optics is their achromatic character: the focal length does not depend on the energy and the energy can be varied over a wide range without modifying the focal point. Conversely, the focal length of CRLs or FZPs depends on the energy.

### 3.2. Scanning XRD Microscopy

The availability of focused beams quickly paved the way for scanning X-ray microscopy. It is generally the sample that is moved in front of the beam; the spatial resolution is essentially dictated by the size of the beam. Scanning X-ray microscopy very quickly followed the introduction of beamlines that could focus the beam [62]. We will limit ourselves here to diffraction maps, but there are many applications for spectroscopic maps [63] that make it possible to visualize, for example, the spatial distribution of an element in a heterogeneous sample. Laue diffraction (white beam) has the very great advantage of not requiring the rotation of the sample, which is always problematic with beams where the size is less than the confusion sphere of the goniometer. From the early 2000s, 2D orientation and deformation maps were produced on thin films by Laue micro-diffraction [16,64], demonstrating the potential of the method (excellent angular resolution and associated deformation at submicron spatial resolution). Despite its many advantages, Laue diffraction has the disadvantage of being sensitive only to the deviatoric part of the strain tensor. This limitation can be circumvented, either by making a monochromatic measurement at the same place [64] or by determining the energy of at least one diffraction spot [65,66]. The extension to three-dimensional mapping has been demonstrated [67] but has not yet given rise to massive uptake because of the difficulty of the experimental set-up and the rapidly prohibitive measurement time. The measurement time associated with deformation mapping is a major parameter that conditions the use of these approaches by many users. The first Laue microdiffraction maps [64] were limited by the reading time of the CCD detector (a few seconds, typically). Now, the use of sCMOS or hybrid pixel detectors allows reading times of the order of a millisecond, and it is the scattering power of the sample itself that sets the counting time. Another limitation to measurement time is the time associated with sample positioning and data transfer. This time demand can quickly become prohibitive (typically, 1 s). A recent development consists of carrying out a continuous scan of the position of the sample and recording the images of the detector “on the fly” [17,68]. The reduction in the time required to perform a mapping is impressive (from 11 h to 7 min for 40,000 points). However, in the monochromatic case, it is necessary to make a map for each angular position inside the rocking curve of the crystal. This type of rapid “on-the-fly” measurement is currently undergoing very significant development and is becoming the norm. We are approaching real scanning microscopy, which is also very useful for quickly positioning oneself within a micro or nanostructured sample. The management of the large flows of data, generated by this type of measurement, deserves special attention, especially since most synchrotrons are evolving toward more brilliance, which will allow even faster measurements. Processing this data to generate an orientation, deformation, etc. map still requires significant time. Significant progress is expected in the coming years on this front. Artificial intelligence—even if the expression is starting to be a bit overused—is certainly an interesting path.

Unlike in the case of scanning electron microscopy, X-ray microscopy uses the scanning of the sample in front of the beam. However, there are situations where the vibrations generated by the movement of the sample are not acceptable (mechanical test on a nano-object, for example). Moving the focusing optical system can allow the beam to be scanned over the surface of a fixed sample [68,69]; this is at the cost, however, of an increase in measurement time.

### 3.3. Coherent Imaging by Diffraction under Bragg Conditions

Coherent imaging by diffraction under Bragg conditions (BCDI: Bragg coherent diffraction imaging) means the acquisition of a 3D diffraction pattern in the far-field, under conditions such that the size of the beam is equal to or less than the transverse coherence lengths and is greater than the size of the crystal, followed by algorithmic processing that makes it possible to trace the amplitude ($\rho (\vec{r})$) and the phase ($\vec{G}\cdot \vec{u}$) of the object in direct space. It is, therefore, a lensless imaging method. The possibility of the convergence of the algorithm is linked to the condition of over-sampling [70] of the diffraction pattern (typically, two points at least must be measured per fringe) ensured in practice by a judicious choice of the pitch of the rocking curve and the position of the detector. The term “coherent diffraction” appeared for a time as a pleonasm. It is now accepted and refers to the fact that the “coherent” diffraction pattern does not result from an incoherent sum of intensities from coherent domains. The volume within which the incident wave remains temporally and spatially correlated is defined as a first approximation by three lengths [71]: the temporal or longitudinal correlation length is directly linked to the energy dispersion and is in the order of a micron at 10 keV when using a Si (111) monochromator. The spatial or transverse coherence lengths are proportional to the size of the source seen from the object and are, therefore, all the greater as the source is small and at a great distance. On long lines (100 m) and around 10 keV, they are typically of the order of several tens of microns. The coherence length is always proportional to the wavelength.

The inversion algorithms are based on iterative round trips between Fourier space (the diffraction signal) and direct space (the crystal). Initially developed for electron microscopy, these algorithms [72,73] are based on the application of constraints in the two spaces. In practice, the constraint in Fourier space is the measured signal and the constraint in direct space is a finite support estimated from the Patterson function. Each reconstruction is associated with the set of random phases that are used initially, and the final result is an average of the best reconstructions.

Since the pioneering work of Robinson [19,44], BCDI has made considerable progress and is becoming a standard-use strain field imaging technique. The convergence of the algorithms toward a “credible” reconstruction is much better. This is due to the improvement of algorithms that now consider the partial coherence of the incident beam [74] or the effects of the refraction and absorption of the beam in the crystal [75]. In addition, great efforts have been made to optimize the mechanical stability of the experimental assemblies. Finally, the appearance of hybrid pixel detectors [21,22,23] that are real photon counters has revolutionized the field. It is no longer necessary to correct the data; the acquisition time of a rocking curve is now around 5 min (previously, around an hour with CCD detectors) which imposes much less drastic constraints on stability. Another major development in the field is the standardization: (i) of the inversion programs used (we can cite the code developed at ESRF by Vincent Favre-Nicolin [76,77], which is on the way to becoming a standard; (ii) criteria for evaluating the spatial resolution [78] or the quality of the reconstructions [79].

Thus, BCDI is entering a phase of maturity that makes it an essential tool for imaging the displacement field in three dimensions, with a displacement resolution of the order of a picometer [80]. As for the spatial resolution, this is directly related to the extent of the reciprocal space in which one can measure a signal. Today located between 10 and 5 nm, it is not completely unreasonable to imagine going down to 1 nm or even to atomic resolution with the new synchrotron sources. The maturity of the method now allows it to be used *in situ* or *operando* during mechanical tests [52], reactions under gas [51,81,82], during electrochemical charging [50], etc. The provision of new synchrotron sources, such as ESRF-EBS, is a huge opportunity for BCDI because the reduced size of the source in the horizontal plane will greatly increase the coherent flux. This will allow the study of smaller objects, to improve the spatial resolution, or to improve the temporal resolution (we can hope for a factor of 100).

Bragg coherent X-ray diffraction imaging is a full-field method that requires an isolated object at least in Fourier space. Thus, it is possible to study micron or sub-micron grains in a polycrystal, provided that their diffraction pattern can be isolated [83,84]. On the other hand, in a bulk material, when it is the beam that defines the diffracting domain, it is necessary to resort to another approach: ptychography. Initially developed in electron microscopy, this technique consists of acquiring multiple coherent diffraction patterns from partially overlapping beam positions [85,86]. The redundancy of the collected information ensures very good robustness for the inversion process and makes it possible to find the incident wavefront [87]. Thus, ptychography in direct beam has become a reference method for the three-dimensional imaging of density in a material [88,89]. In Bragg geometry, despite pioneering work [49,90], the method is still in its infancy, perhaps because of the very restrictive mechanical stability constraints it imposes. During BCDI measurements, it is now common practice, at the start of the experiment, to use ptychography on a reference object (often a Siemens star) to characterize the wavefront. Very recently, a method has been developed to circumvent the limitation of BCDI in the study of crystals with dimensions smaller than the size of the coherent beam [53]: this involves cutting a crystal of micrometric dimensions using an ion beam (FIB: focused ion beam) and imaging this fragment in BCDI. The price to be paid is the local destruction of the sample, but it is, thus, possible to make an extremely detailed analysis of the local distribution of the dislocations.

### 3.4. Dark Field Imaging

X-ray microscopy, in the sense that it is understood for its sister techniques “electron microscopy” and “optical microscopy”, is a full-field microscopy that uses an objective lens between the sample and the detector. The most convincing results have been obtained using refractive lenses [91]. The spatial resolution is limited by the quality of the lenses and is, today, around 100 nm. The use of MLLs should make it possible to drop down to a few tens of nm.

In full-field microscopy, it is possible to obtain an image that is sensitive to local deformations in the crystal by placing oneself in diffraction conditions [18]. The dark field image that is thus obtained shows a contrast that evolves along the rocking curve. Developed by the Danish team from DTU (Technical University of Denmark) on the ID06 beamline of the ESRF, this dark-field microcopy has quickly reached an advanced stage that makes the technique very interesting for many scientific scenarios. Thus, 3D maps of orientation and strain have been measured in the emblematic ferroelectric compound BaTiO_3_ [92], with spatial, orientation and deformation resolutions of 100–200 nm, 1 mrad and 10^−5^, respectively.

Dark-field microscopy has the advantage of being a full-field technique; therefore, it is intrinsically faster than scanning methods and is more suitable for *in situ* measurements for which temporal resolution is important. However, this statement should perhaps be tempered, insofar as a complete 3D mapping in deformation requires an angular scan of two angles for the sample and of the angular position of the detector (which also implies a translation of the objective lens).

The rise of new synchrotron sources will allow, as for other mapping techniques, the accelerated development of dark-field microscopy, which will benefit from the increase in brilliance to further decrease the acquisition time.

## 4. Application Examples

### 4.1. Coherent Bragg Imaging in Nanostructures

**Figure 4:**
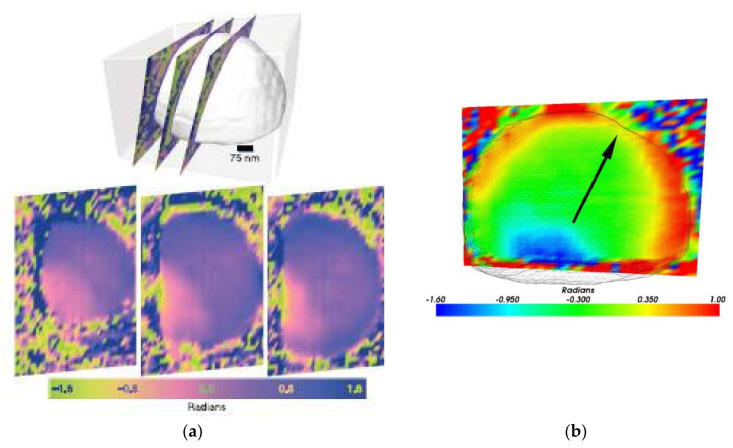
(**a**) Phase mapping inside a lead particle for 3 parallel sections that are 138 nm apart. The phase variations come from the deformation field generated by the contact forces at the interface with the substrate. (**b**) Phase anisotropy approaching free surfaces; the arrow indicates the direction [111] of the scattering vector [19,75]. Reprinted from [19,75]. Reprinted figure with permission from [75]. Copyright (2007) by the American Physical Society.

As we have seen previously, BCDI has the immense advantage of obtaining the image of an object in 3 dimensions from a 3D measurement of the diffraction peaks. In the case where a single diffraction peak is measured, in addition to the shape of the sample, information is obtained on the component of the displacement field $\vec{u}$ along the direction of the scattering vector $\vec{G}$ of the diffraction peak. Thus, in the very first experiment using BCDI [19], the 3D measurement of the Bragg spot 111 coming from a lead particle of 750 nm in diameter made it possible to determine the component of the displacement field in the direction [111] inside the particle (Figure 4a).

The lead particle, obtained by dewetting a thin film deposited on the native oxide of a silicon substrate, does not show strong deformation. The maximum value of the displacement field along the direction [111] is 0.5 Å. No defect is observed inside the particle; the residual displacement field is interpreted as resulting from the contact force at the interface with the substrate. The analysis of the displacement field shows the sensitivity of the method, since, once the refraction corrections were applied [75], it was possible to reveal the anisotropy of the surface stresses (Figure 4b).

To obtain complete information on the displacement field, it is necessary to resort to the measurement of several diffraction peaks. The difficulty lies in the ability to stay on the same nano-object when it is reoriented several times, to satisfy the Bragg conditions of the different reflections. This was achieved for the first time on a rod-shaped ZnO nanocrystal [93]. The measurement of 6 diffraction spots made it possible to determine the complete strain tensor in a sample that, nevertheless, did not present any defect or significant strain field.

**Figure 5:**
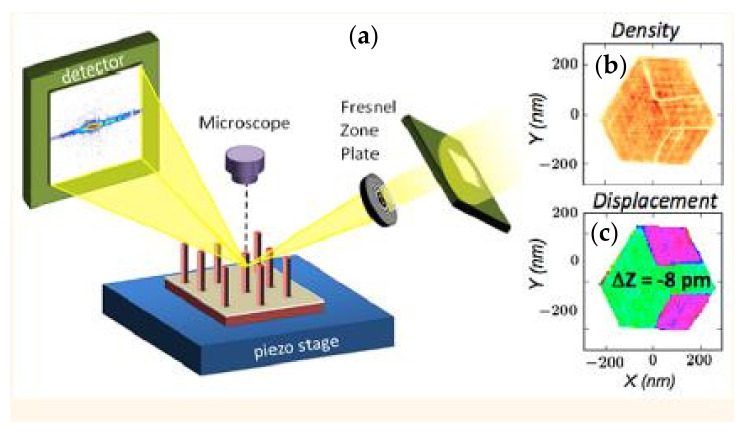
Principle of a 3D diffraction measurement at the mid-height of a GaN nanowire (**a**) images obtained at mid-height of the nanowire giving the density (**b**) and the component of the displacement field along the axis of the nanowire (**c**); the different colors indicate the presence of two inversion domains [80]. Reprinted from [80].

The ability of this technique to image crystalline defects has since been widely demonstrated. Thus, the configuration of inversion domains in GaN nanowires (Figure 5) could be studied from the measurements of 5 Bragg peaks [80]. The growth of the nanowires was achieved by organometallic vapor phase epitaxy on the (0001) surface of a sapphire substrate. Since the nanowires are all oriented in the same way, it is necessary that they are sufficiently far apart from each other to facilitate the diffraction measurements of a single nanowire. The hexagonal wires have a diameter of about 600 nm for a height of 4 microns and are spaced about ten microns apart. The focusing of the beam produced using FZP made it possible to analyze a portion at the mid-height of the wire. The internal structure that is, thus, revealed highlights the complex arrangement of inversion domains. The determination of the three components of the displacement field was carried out with a spatial resolution of 6 nm and an accuracy of 1 pm along the c axis and 4 pm in the (0001) plane. The unambiguous determination of the polarity of the domains made it possible to establish that the domains terminated by a Ga plane were shifted, with respect to the domains terminated by an N-plane, by (c/2+8) pm (Figure 5) along the axis c and 0 pm in the (001) plane. These experimental values were subsequently confirmed by ab initio calculations [94].

**Figure 6:**
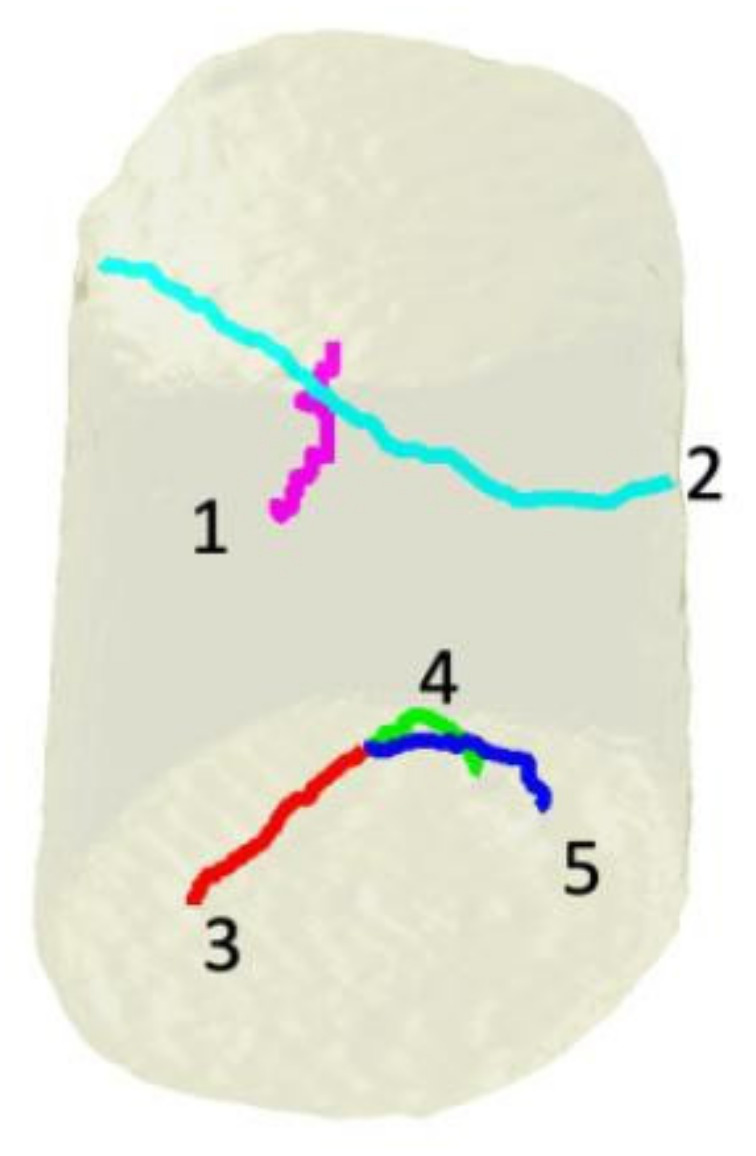
The 3D arrangement of 5 dislocation lines in a portion of a tungsten crystal. Image made from a combination of 6 independent reconstructions of (110) Bragg peaks [53]. Reprinted from [53].

However, the three-dimensional imaging of plane defects is not the only possibility offered by this technique. Thus, dislocations can be identified and imaged in 3D inside a crystal. Even if the current resolution does not allow going below 5 nm and, therefore, makes it impossible to analyze the core of the dislocations, the displacement field generated at long range by the dislocations makes their identification obvious. Recently, a complex configuration of 5 dislocation lines in an isolated part of a tungsten crystal was highlighted [53]. Several defects were deliberately introduced by indentations in the tungsten; then, a portion of the monocrystal of approximately (500 nm)^3^ was isolated by FIB. The diffraction measurement on six (110) planes then made it possible to identify a complex arrangement of five dislocations (Figure 6). The Burgers vectors of each arm could be identified using the three components of the displacement field determined in 3D in the crystal. In the end, the level of comparison of the experimental values of the complete strain and rotation tensors with the theoretical values calculated from the real configuration of the dislocations is remarkable.

**Figure 7:**
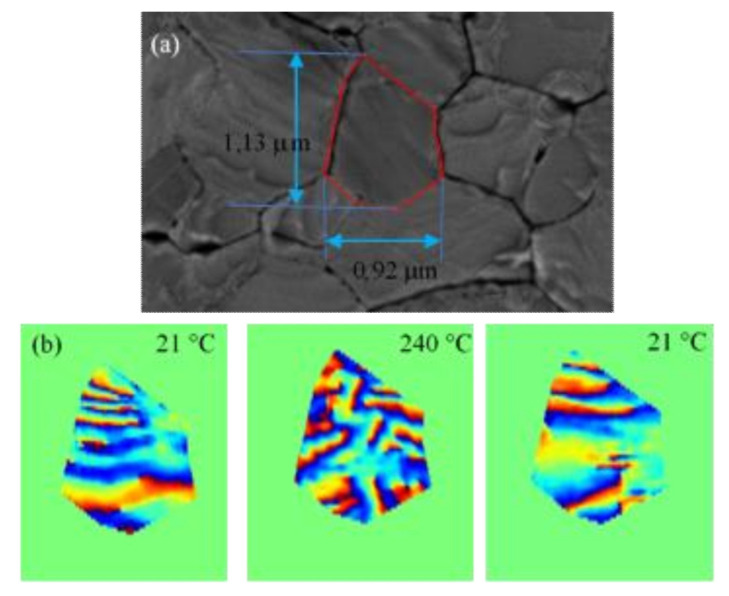
(**a**) Scanning electron microscopy image of a polycrystalline gold film; the measured grain is indicated in red. (**b**) Mid-height section of the displacement field as a function of temperature [83]. Reprinted from [83].

Even though a large majority of BCDI experiments have been performed on isolated crystals, it is nevertheless possible to obtain a 3D image of an individual grain belonging to a polycrystalline film. This requires that the grains are sufficiently misaligned relative to each other. Thus, the diffraction peaks are naturally separated in the angular space and the individual measurement of a diffraction spot can be accomplished. Experiments of this kind have been performed on a polycrystalline gold film deposited on glass (Figure 7a). They were coupled with a thermomechanical loading that uses the difference in the coefficient of thermal expansion between gold and glass to apply compressive stress in the gold film when the sample is heated [83]. Temperature increments of 25 °C, i.e., 0.03% in compression, were applied up to a temperature of 250 °C and the same increments were made during cooling. Grain realignment in the beam is required at each increment, due to furnace expansion, to which must be added the temperature stabilization time. Including the measurement time, each temperature step took 1 h. A large anisotropy of the component of the displacement field perpendicular to the film was observed in the grain. This anisotropy decreases during loading and returns almost to its initial state during unloading (Figure 7b). This behavior could be attributed to the interaction between the grains related to the orientation of the neighboring grains, as determined by EBSD and Laue micro-diffraction. The comparison of the mechanical behavior of the grain with that of the whole film confirmed the importance of localization effects in these polycrystalline films.

**Figure 8:**
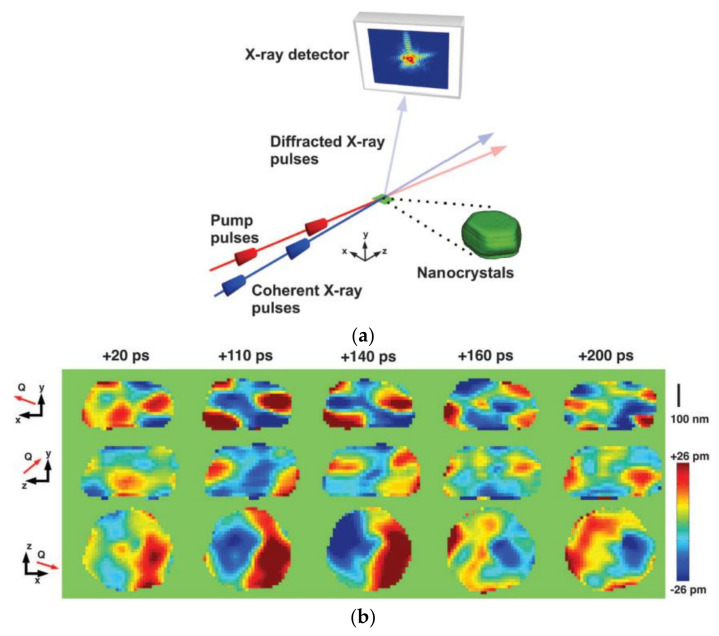
(**a**) Principle of ultrafast BCDI measurement. (**b**) Orthogonal section passing through the center of the particle, showing the composition of the displacement along the scattering vector Q as a function of time [95]. Reprinted from [95].

BCDI has also been used to follow the evolution of a gold nanoparticle at the scale of ten picoseconds during ultrafast pump-probe experiments [95]. To do this, the diffraction is carried out using an ultra-bright pulsed source of the XFEL type, triggered with a modulable delay ranging from 10 to 500 ps after an optical pulse, which excites the vibration modes of the crystal (Figure 8a). The contribution of this experiment is, on the one hand, to have been able to measure the signal coming from a particle and not from a set of particles that systematically present size and shape dispersions. On the other hand, the imaging of the displacement field, resolved in 3D at a scale of 50 nm and temporally at a scale of ten picoseconds, made it possible to identify modes of shear vibration that are impossible to observe if we only measure the barycenter of the spot because they do not give rise to the displacement of the Bragg peak, just to a modification of its shape. Indeed, Figure 8 clearly shows that the expanding regions become compressed, and vice versa, when the time difference with the laser pulse increases. This spatial and temporal inversion of the deformation states clearly indicates the presence of a higher-order shear vibration mode, compared to the simple breathing mode of the particle.

### 4.2. Imaging of Deformations by Scanning XRD Microscopy

Scanning XRD microscopy is particularly applicable to surface-structured (solid or thin-film) crystals, for example, Si substrates with through-vias [96,97,98], semiconductor films (Si, Ge, GaN, InGaN, etc.) with surface defects [99,100,101,102], semiconductor micro-bridges [103,104], heterostructures [105], (micrometric poly-)crystals [106,107] or nano-membranes [108,109].

The advent of two-dimensional (2D) detectors engenders the collection of 5D data obtained by mapping a sample spatially in two directions and at different angles of rotation [17,103]. The measurement of a 3D Bragg hkl reflection (2D images of the detector, collected at different angles of rotation), and the extraction of the maximum or center of mass of the diffraction peak(s) for the different positions (x, y) of the cartography, makes it possible to locally determine the deformation field and the orientation of the (hkl) planes measured. The measurements, therefore, generate 5D datasets with a high number of images (several hundred Gbits of data), which is a challenge in terms of data management, storage, and processing. A software called XSOCS (XSOCS: X-ray strain orientation calculation software) is notably available (https://gitlab.esrf.fr/kmap/xsocs, accessed on 4 December 2022) for the processing of this data. The technique (K-mapping) offers a resolution in terms of strain field Δε = Δa/a = 10^−5^ (where a is the lattice parameter) and in terms of crystallographic orientation, the variation or tilt of the crystallographic planes of 0.001° [17].

Unlike the ptychography technique, which requires the use of a coherent beam and inversion algorithms, only the maximum or center of mass of the diffraction peak needs to be measured accurately at each point. Therefore, the technique is much more stable regarding the possible fluctuations (intensity and shape) of the beam, but the spatial resolution is limited to the size of this beam, whereas for ptychography, the resolution is much better and is theoretically limited by the wavelength of the beam.

**Figure 9:**
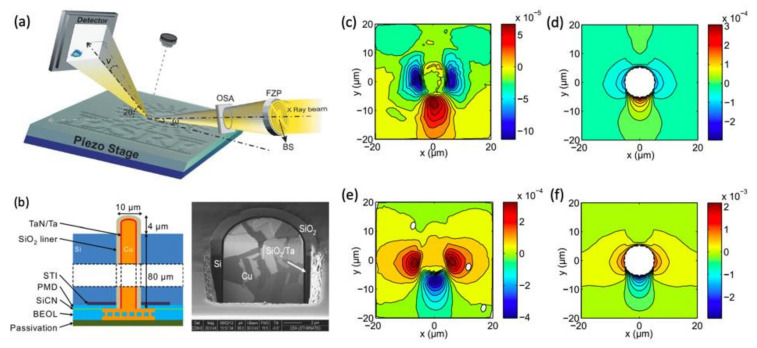
(**a**) Experimental scanning XRD microscopy device on the ESRF beamline ID01 [17]. (**b**) Schematic view and FIB/SEM section of the measured sample. (**c**–**f**): Two-dimensional measurements of the deformation field of Si along the [335] direction, around a Cu via at room temperature (**c**): experiment and (**d**): simulation) and during the *in situ* annealing at 400° C (**e**): experiment and (**f**): simulation) [96]. Reproduced from [96] with the permission of AIP Publishing.

Being non-destructive, the technique can be applied *in situ* or even *operando*, such as during annealing [96] or mechanical testing [68]. Figure 9 shows the experimental setup used on the ESRF ID01 beamline. The beam is nano-focused (350 nm (horizontally) × 200 nm (vertically)) using a Fresnel zone plate (FZP) and the diffraction signal is measured with a 2D detector. With this nano-beam, it was possible to map the deformation field in Si around a via (a metalized hole) in the Cu (see sample diagram in Figure 9b) [95]. The strain field was measured along the [335] crystallographic direction of Si at room temperature (Figure 9c) and *in situ* during annealing at 400 °C (Figure 9e). These measurements show that the strain field, ε_335_, in Si remains below 4 × 10^−4^. An inversion of the sign of the strain field is observed between the two temperatures, which agrees with the difference in thermal expansion between Cu and Si. Simulations by the finite element method have been carried out (see Figure 9d,f). They are in good agreement with the experiment and show the need to consider the plasticity of copper in the modeling.

**Figure 10:**
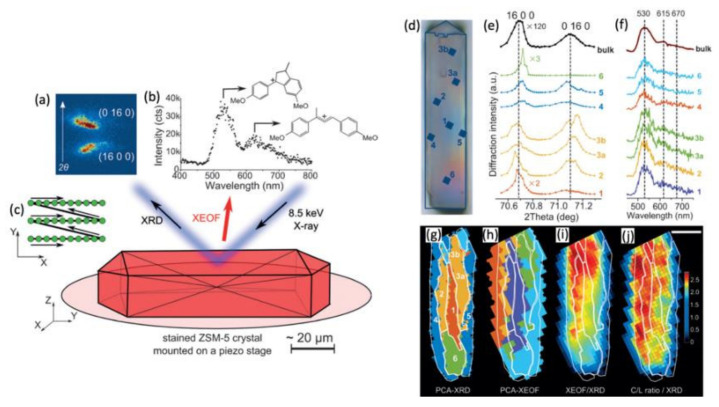
Combined μ-XRD and μ-XEOF (X-ray excited optical fluorescence) experiment when measuring a zeolite micro-crystal. (**a**) A 2D image of the detector with the two diffraction peaks. (**b**) XEOF spectrum, detected with a UV/Vis spectrometer. (**c**) An x-y scan to acquire the spatially resolved μ-XRD and μ-XEOF intensity maps. (**d**) Optical micrograph of the sample. (**e**) Diffractograms and (**f**) XEOF spectra, measured at different positions on the sample. (**g**–**j**) Principal component analysis of μ-XRD and μ-XEOF measurements and the combination of measurements. The scale bar corresponds to 20 μm [106]. Reprinted from [106].

The scanning XRD microscopy technique can be combined with other techniques, such as X-ray fluorescence [110], to obtain a chemical map where fluorescence or optical luminescence is excited by X-rays (XEOF or XEOL) [106]. Figure 10a–c shows the combined use of μ-XRD scanning microscopy and micro-focused X-ray beam excited optical fluorescence (μ-XEOF) for the measurement of a zeolite crystal. With a 500 nm X-beam, it was possible to locally measure two Bragg reflections of the zeolite crystal (16 0 0) and (0 16 0) (see Figure 10a–d), while collecting the XEOF signal (Figure 10f) with an optical fiber positioned a few hundred microns from the sample. Correlations between the structural (μ-XRD) and optical (μ-XEOF) signals could be made, thanks to principal component analysis (PCA–see Figure 10g–j), showing the phase anisotropy of the crystal in connection with a different reactivity.

**Figure 11:**
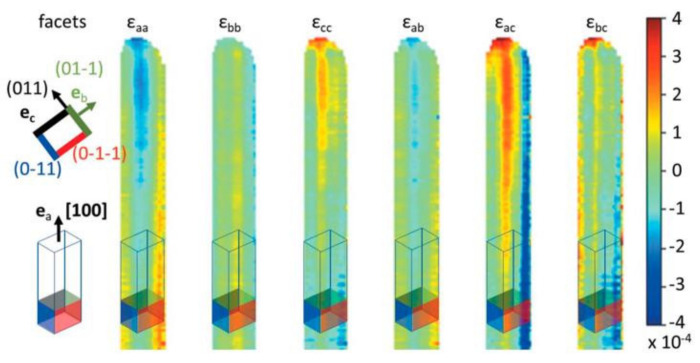
Mapping of the components of the strain tensor expressed in a base (a, b, c) whose vectors are aligned with the facets of the VO_2_ microwire. Facets perpendicular to the c, b,-c, and -b directions are colored black, green, red, and blue, respectively. This orientation is superimposed on the maps to guide the reader [112]. Reproduced from [112] with the permission of the International Union of Crystallography.

Other works have shown that it is possible to locally extract not only the strain field or the misorientations of the measured crystallographic planes but also the composition field [101,111] for a bi-material (for example, a thin SiGe layer), thanks to the measurement of two Bragg reflections. In this case, it is necessary to formulate hypotheses on the bi-material, such as for example assuming a composition verifying Vegard’s law and bi-axial stress. To access the complete strain tensor **ε**, it is necessary to measure three non-coplanar reflections. Recently, it has been shown that the set of components ε_ij_ of the strain tensor can be determined from two Bragg reflections [111], if the material incompressibility condition is imposed. This method was used to reveal the surface stress-induced strain on the edges of a monocrystalline micrometer-long VO_2_ wire [112] (see Figure 11). All microwire strain tensor components were measured up to an absolute minimum value of 10^−4^, over a microwire projected area of 8 × 14 μm^2^. With a beam-defined spatial resolution of 150 × 150 nm^2^, the measurement took only 2.5 h.

The development of focusing systems makes it possible to reduce the size of the beams and, therefore, to improve the spatial resolution. Recently, beam size of the order of 10 nm [113] and even lower (8.4 nm × 6.8 nm) [114] has been obtained, thanks to Laue lenses. The development of nano-focused beams opens an important field for this technique. It should be noted that the use of nano-focused beams requires the excellent stability of the beamline (thermal and mechanical stability, etc.) and very good motor precision (use of piezoelectric motors, for example). Care must also be taken to stay on the measured area when rotating the sample (i.e., when changing the angle of the rocking curve). The typical sphere of confusion of state-of-the-art diffractometers is on the order of 10 μm over a full rotation. This can lead to parasitic displacements or drifts of the sample relative to the beam during the rotation of the sample, these drifts being caused, for example, by an error in the eccentricity of the axis of rotation, which can be greater than the size of the beam or of the measured structures. It is therefore important to determine *a priori* or *a posteriori* these drifts (characterization of the eccentricity of the goniometer, crystalline markers on the sample, etc.). Future improvements in terms of X-ray optics and motor positioning via the development of interferometers [115], for example, should allow scanning strain imaging with a resolution approaching a few nm [116,117].

The possibility to perform “on the fly” measurements (i.e., with continuous movement of the sample’s translation motors) has made it possible to drastically reduce the measurement time thanks to the elimination of the dead time, linked in particular to the movement of the motors [17,118,119]. With the quasi-elimination of the dead time and the development of detectors able to operate in the kHz regime, the scanning speed will, in the long term, be limited by the counting statistics and, therefore, the brilliance of the source and/or the scattering power of the sample. This technique will benefit from the multiple third-generation synchrotron upgrade projects underway or planned in the coming years and is likely to become a possible routine analysis technique for structured crystalline materials.

### 4.3. Laue Micro-Diffraction Applied to the Mechanics of Nano and Micro-Objects

Laue microdiffraction using a white beam is very sensitive to crystal orientation and defects such as geometrically necessary dislocations (GNDs). Thanks to the polychromatic nature of the incident X-ray beam, a multitude of diffraction spots can be measured simultaneously without any rotation of the sample, unlike monochromatic X-ray diffraction, where the incident angle and the diffraction angle must be adjusted for each Bragg reflection. This makes Laue microdiffraction very attractive for *in situ* experiments, in particular, for micro- and nano-mechanical studies for which every additional movement must be avoided, in order to avoid the generation of harmful vibrations. Due to these constraints, typical *in situ* experiments are performed with the focused X-ray beam illuminating a single point on the sample, thus limiting the information obtained to one position on the structure being studied. To map the deformation of a mechanically loaded nanostructure, Leclere et al. have developed a new method that scans the X-ray beam over the sample surface, like the electron beam in an SEM, by moving the Kirkpatrick-Baez mirrors used to focus the polychromatic X-ray beam incident in the horizontal and vertical directions [69].

In the following, some examples of *in situ* micro- and nano-mechanical tests, in combination with Laue microdiffraction, are presented. In recent years, various tools have been designed for the in situ mechanical testing of micro- and nanostructures, including *in situ* indenters for the micro-compression testing of metal pillars [120,121,122] and an *in situ* atomic force microscope [123].

**Figure 12:**
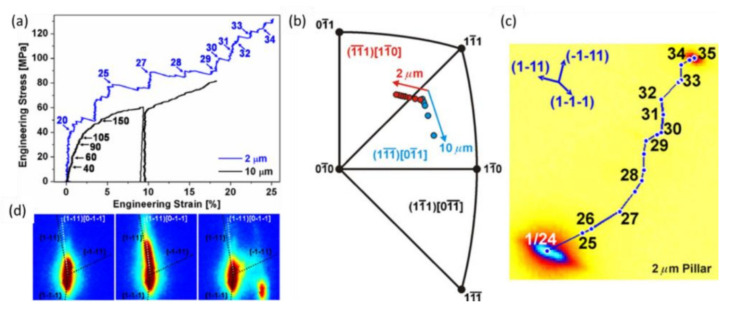
(**a**) Stress-strain curves for an FIB-etched Au micro-pillar during compression. The arrows indicate the corresponding Laue microdiffraction pattern recorded during the mechanical test. (**b**) Inverse pole figure showing the evolution of the real vertical crystalline axis during the compression test. (**c**) Trajectory of the Au Laue peak on the detector, with the same numbering as that indicated in (**a**). (**d**) Intensity distribution of the Laue peak at a load of 0 MPa (#0), 40 MPa (#20) and 77 MPa (#25), respectively [120,127]. Reprinted figure with permission from [120]. Copyright (2007) by the American Physical Society.

The stress-strain curves of the compression of $\left[4\bar{6}3\right]$ oriented and FIB-machined gold pillars with diameters of 2 and 10 μm are shown in Figure 12a, where the numbers indicate the corresponding Laue microdiffraction patterns recorded during the *in situ* mechanical tests. By depicting the vertical crystal orientation of gold in the inverse pole figure (Figure 12b), the slip systems activated in the 10 μm pillar were identified to be the same as predicted, i.e., those with the highest Schmid factor. However, for the 2 µm diameter pillar, unexpected slip systems were found. The path of the satellite peak on the detector (from the deformed part of the micro-pillar) revealed that the unexpected rotations were composed of several activated slip systems (Figure 12c), which finding was confirmed by observing the slip marks by SEM imaging. This unexpected behavior was attributed to the strain gradients present in the pristine pillar, which were inferred from the asymmetric shape of the diffraction peak (Figure 12d). During micro-compression testing, there is friction between the flat punch of the indenter and the upper end of the micro-pillar. This friction hinders the lateral movement of the structure and induces additional stresses that generate additional bending components in the uniaxial test. Thus, strain gradients are created, and the central part of the crystal rotates so that the slip planes and the slip direction approach or move away from the loading axis. The effect of stresses has been studied in detail in a series of studies by Kirchlechner et al. [122,124,125] and was recently reviewed by Robach et al. [126].

**Figure 13:**
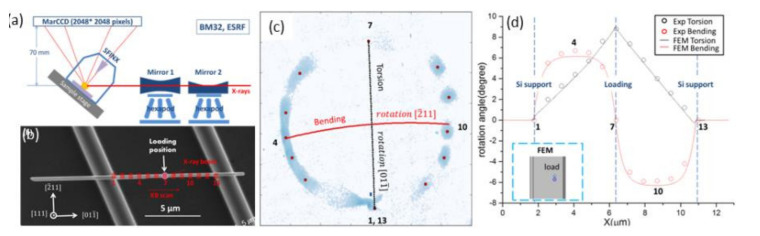
(**a**) Schematic representation of the experimental setup on the BM32 line. (**b**) Scanning electron microscopy image of a self-suspended Au nanowire before deformation. Measurement positions along the nanowire during KB scans are marked with rectangles and the scan direction is indicated by the arrow. (**c**) Magnified view of the area around the Au 222 diffraction peak for a tip displacement of 1100 nm. The expected movements of the Laue spot on the detector for pure vertical bending (rotation around the Au direction $\left[\bar{2}11\right]$) and pure torsion (rotation around the Au direction $\left[01\bar{1}\right]$) are marked respectively in red and black lines. (**d**) Bending and torsion profile along the suspended part of the nanowire deduced from the integrated Laue micro-diffraction diagram shown in (**c**) and calculated by simulations by the finite element method, considering a force of 2.1 µN and misalignment of the SFINX tip with respect to the center of the nanowire by 57 nm [129]. Reproduced from [129] with the permission of AIP Publishing.

*In situ* three-point bending experiments on individual metal nanowires placed across micro-trenches (see Figure 13a,b) were conducted, using an *in situ* AFM [128,129]. The elastic and plastic deformation was followed *in situ* by Laue microdiffraction and by scanning the X-ray beam along the charged nanowire, using the KB scanning approach. During the loading of the nanowire, the Laue spots move on the detector. The Au 222 Laue spot integrated along the mechanically loaded nanowire is displayed in Figure 13c. While pure bending causes a vertical movement of the Laue spot on the detector, pure torsion causes a horizontal motion of the spot. The circular motion of the Laue spot indicates the presence of both the bending and twisting of the nanowire, which was confirmed by finite element method simulations, considering a misalignment of the loading point of about 60 nm with respect to the center of the nanowire.

**Figure 14:**
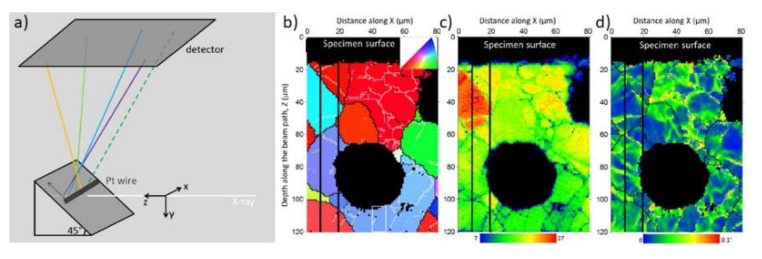
(**a**) Schematic illustration of the differential aperture X-ray microscopy (DAXM) approach. (**b**) Crystallographic orientation, (**c**) the number of indexed points, and (**d**) local misorientation map showing the microstructural details around a graphite nodule (black circular blocks) characterized using polychromatic DAXM. The colors in (**b**) represent the crystallographic orientation along the direction normal to the sample (see inset in (**b**)). On the maps, cell walls of dislocations with misorientation angles in the range of 0.1–1°, 1–3°, and > 3° are shown as thin white lines, thin black lines, and thick black lines, respectively. The individual black pixels in the array away from the nodules are unindexed pixels [130,131]. Reprinted from [131].

The high-energy polychromatic X-ray beam used for Laue microdiffraction penetrates deep into the material; therefore, a signal from the full penetration depth is recorded. To obtain depth-dependent information, 3D Laue microdiffraction (also known as differential aperture X-ray microscopy (DAXM)) was developed: a wire of approximately 100 µm in diameter is scanned parallel to the sample surface at a distance of a few hundred micrometers, which obscures parts of the diffracted X-ray beam as shown schematically in Figure 14a [67]. From trigonometric considerations and from the intensity variation of the Laue diffraction spots as a function of the position of the wire, the depth of the diffracting grain can be determined with a resolution of approximately 500 nm. The angular resolution is determined by the size of the pixels and the distance between the sample and the detector, which gives approximately 0.01°.

Figure 14 shows an example of DAXM on ductile iron, which is a composite material model consisting of graphite nodules embedded in a metallic matrix [130,131]. The grain orientation versus depth map around a graphite nodule with a size of 50 μm is shown in Figure 14b, revealing dislocation stacks with low misorientation angles. It appears that most of the deformed grains contain grain sub-boundaries with misorientation angles less than 1°, with dislocations that are organized in a cellular structure. The orientation variation inside the cells was determined by calculating the local mean misorientation (KAM: kernel average misorientation) for each pixel displayed in Figure 14d.

Although Laue microdiffraction using a white beam is very sensitive to crystal orientation, it does not provide access to absolute strain but only to deviatoric strain within the crystal. This deficiency comes from the fact that the energy of the diffracted X-rays is unknown. Recently, energy-dispersive 2D pixel detectors, originally developed for particle physics, have been introduced to the synchrotron community. These cameras make it possible to measure the energy of diffracted X-rays and, thus, provide access to absolute deformation, with resolutions of around 1% [65,132]. Another technique for measuring absolute deformation is the so-called rainbow method, which relies on the introduction of a monochromator crystal, upstream of the sample, to filter preselected energy. Due to this energy filter, the intensity of some Laue spots decreases, allowing the diffraction spots to be correlated with the energy of the scattered X-rays. This technique gives an energy resolution of the order of 1 eV and, therefore, a deformation resolution of about 10^−4^ [66].

The current upgrade of third-generation synchrotrons to extremely brilliant sources with reduced source sizes and less divergent X-ray beams will increase the available flux densities. In addition, new detectors based on sCMOS technology will significantly reduce dead time. These improvements will not only speed up current experiments but will also provide new opportunities, such as the study of smaller nanostructures, the nanostructures of materials containing lower atomic number elements (which diffract less) or experiments resolved in time, for example, for fatigue measurements. Future energy-dispersive detectors with larger active areas will eventually allow the obtaining of crystal orientation matrices, as well as the full strain tensor in a single measurement. A further improvement in energy resolution, which currently stands at about 1%, will open new perspectives for strain-resolved white-beam Laue microdiffraction upon the application of external stimuli.

## 5. Conclusions

The imaging of deformations with X-rays has continued to progress in recent years. Three techniques now make it possible to map deformations in two or three dimensions, with a spatial resolution that ranges from approximately 100 nm to 5 nm: (i) full-field microscopy in the dark field (resolution 100 nm); (ii) scanning diffraction microscopy (50 nm resolution); (iii) coherent imaging under Bragg conditions (5 nm resolution). These advances are based on: (i) the continuous improvement of X-ray-focusing optics associated with the implementation of long beamlines; (ii) the development of two-dimensional hybrid pixel detectors with high dynamic range, high reading speed, and low noise level; (iii) the implementation of “on the fly” measurements (continuous movement of the motors) making it possible to significantly reduce acquisition times.

The new synchrotron sources that are being set up around the world (for example ESRF-EBS in 2020) will allow a very significant increase in brilliance and coherent flux. These new instruments should allow the three-dimensional imaging of deformations at rates fast enough to follow *in situ* the evolution of a material under stress or during a transition. The reduced size of the source will also allow very significant gains in spatial resolution. Processing huge amounts of data is one of the big challenges to address in the near future.
